# WS_2_ Nano-petals and Nano-bristles Supported on Carbon Nanotubes for Electron Emission Applications

**DOI:** 10.1038/s41598-019-39605-4

**Published:** 2019-03-06

**Authors:** Tamie A. J. Loh, Ying Jie Ooi, Daniel H. C. Chua

**Affiliations:** Department of Materials Science and Engineering, National University of Singapore, 7 Engineering Drive 1, Singapore, 117575 Singapore

## Abstract

Atomically thin WS_2_ nano-petals and nano-bristles were synthesized on vertically aligned carbon nanotubes (CNT) via magnetron sputtering at room temperature. The formation of the nano-petal morphology requires reaching a critical threshold in sputter deposition time, below which an amorphous film of WO_3_ is obtained instead. Increasing the deposition time past a second threshold results in a change to the nano-bristle morphology. Both WS_2_ nano-petals and nano-bristles were able to significantly enhance the electron emission of properties. The lowest turn-on voltage measured was to be 295 V and 355 V for the nano-petals and nano-bristles respectively, versus 425 V for pristine CNTs. The variation in the turn-on voltage is due to the electrical contacts at the interface between the different WS_2_ structures, which induces current saturation at high emission currents. These results demonstrate that 2D WS_2_ layers can be synthesized without the need for chemical routes and high growth temperatures if an appropriate template is employed.

## Introduction

Since the discovery of graphene, layered inorganic analogues of the material have gained significant attention from the scientific community due to their interesting properties and wide potential applications in various nanoelectronic devices. Tungsten disulphide (WS_2_), a typical layered transition metal dichalcogenide (TMD), is composed of individual planes of W atoms with 6-fold coordination symmetry that are hexagonally packed between two trigonal layers of S atoms^1,2^. Due to its unique layered structure and relatively narrow band gap, WS_2_ has found uses in lithium ion batteries^3,4^, supercapacitors^4,5^, sensors^6^, hydrogen production^7,8^, and photovoltaics^9^. With their high electrical conductivity and mechanical robustness, 2-dimensional (2D) TMDs such as WS_2_ are also promising candidates for next generation field emitters. However, though a number of field emission studies has been conducted for graphene, research on 2D WS_2_ as potential field emitters remains largely undeveloped. Some early works include the fabrication of WS_2_ nanoflowers by atmospheric pressure chemical vapour deposition (CVD)^10^ at 650 °C, which demonstrated a turn-on field of 6.1 V μm^−1^. Another study synthesized WS_2_ nanotubes on Si microspike arrays^11^ with turn-on fields of ~2.6 V μm^−1^, which is comparable to that of vertically oriented carbon nanotubes (CNT).

The practical application of WS_2_ in various fields such as field emission is often limited by intrinsic defects due to the semiconducting nature of the material. One way to overcome this is to combine different materials to form composites or heterostructures, thereby enhancing existing properties and/or inducing additional properties that may be too difficult to achieve within a homogeneous system. Carbonaceous nanomaterials such as graphene and CNT remain the most commonly used materials for hybridization with TMD nanosheets to form composites. This is due to the fact that chemically converted graphene such as GO and chemically grown nanotubes possess abundant oxygen-containing groups that can facilitate the immobilization of other materials grown on their surface, making them promising templates for the preparation of composites^12^. In addition, the unique geometry and high aspect ratio of one-dimensional (1D) CNTs along with their excellent mechanical and electrical properties^13,14^ make them an ideal candidate for electron emitters in vacuum nanoelectronic devices. Field emission performance of CNTs has been widely reported to be enhanced through coating with semiconductor materials such as MoO, ZnO and MgO, due to the formation of Schottky junctions at the interface^15–17^. The introduction of a semiconducting WS_2_ shell on the side walls of CNTs is thus expected to improve the field emission properties of the nanotubes. The TMD coating would also act as a protective layer for the conductive nanotube core that minimizes emission instability due to adsorption or ion bombardment of residual gas molecules.

Various techniques have been used to hybridize CNTs with TMD nanosheets, most of them chemical in nature, and thus require high temperatures and long growth times for production of high quality crystals^18–20^. Such harsh environments often lead to degradation of the CNT structural integrity, which in turn impairs the field emission performance. For obtaining large quantities of nanosheets, solvent-based exfoliation of MoS_2_ and WS_2_ is frequently used. This technique is also promising because it permits the fabrication of composites and hybrids by simple mixing of dispersions of different materials^21,22^. Chemical vapour deposition (CVD), which has been very successful at growing high quality graphene, was recently found to be effective at producing large area MoS_2_ and WS_2_ thin films on insulating substrates such as SiO2, mica and sapphire^23,24^. However, the technique requires high process temperatures in the range of 650–1000 °C and long growth times. Many of these synthesis methods also require more than one step to obtain the final structure, which makes them time-consuming and non-economical. To overcome these limitations, we demonstrate a simple one step physical approach to obtain WS_2_-coated vertically aligned CNTs at room temperature via magnetron sputtering. As opposed to chemical based methods, sputtering offers greater control over the morphology and thickness of the resulting film, and is economical and clean because no external catalysts or solvents were used.

## Experimental

Thin film Fe catalysts were deposited on highly n-doped Silicon (100) substrates using radio-frequency (RF) magnetron sputtering system Denton-Discovery-18. The substrates were cleaned with ethanol and blow-dried using a nitrogen gun before being placed in the sputtering chamber. Sputter deposition was performed at a working pressure of 10^−2^ Torr and RF power of 100 W. The substrates were subsequently transferred in air to the plasma enhanced chemical vapor deposition (PECVD) growth chamber, which was then pumped down to a pressure of ~10^−5^ Torr and heated to 700 °C. C_2_H_2_ and H_2_ gases were used in the growth process at flow rates of 40 sccm and 60 sccm respectively. Growth of multi-walled CNTs was performed for 60 minutes at a pressure of 1.2 Torr and RF power of 100 W. The resulting CNTs were then tip-coated with WS_2_ via RF magnetron sputtering using a WS_2_ target (99.9% purity). Sputter deposition was carried out at room temperature, a low RF power of 40 W, a pressure of 10^−2^ Torr and deposition times of 10, 20, 25, 30, 35, 40, 50, and 60 minutes.

Surface morphologies of the samples were characterized by a Zeiss Supra 40 field emission scanning electron microscope (SEM) using an in-lens secondary electron detector, and a JEOL JEM-3010 transmission electron microscope (TEM) operating at an accelerating voltage of 300 kV. Surface composition was analyzed by x-ray photoelectron spectroscopy (XPS), using a Kratos Analytical Axis Ultra^DLD^ UHV spectrometer with a monochromatized Al Kα x-ray source (1486.6 eV). Core-level XPS spectra were obtained by photoelectrons at a take-off angle of 90°, measured with respect to the sample surface at a vacuum of 5 × 10^−9^ Torr. The C 1 s peak at 284.4 eV binding energy was used as reference to calibrate the spectrum for analysis. Crystallographic data was obtained through a Bruker D8 Advanced Thin Film X-ray Diffraction (XRD) system using a Cu Kα source. The optical properties of as-fabricated samples were characterized using a Horiba MicroRaman HR Evolution System with 514.5 nm excitation wavelength. Field emission studies were conducted using a probe tip setup at room temperature and base pressure of 2 × 10^−6^ Torr. The anode was an electrochemically etched tungsten needle with a tip diameter of 50 μm, positioned 10 μm away from the sample to be tested (cathode).

## Results and Discussion

**Figure 1:**
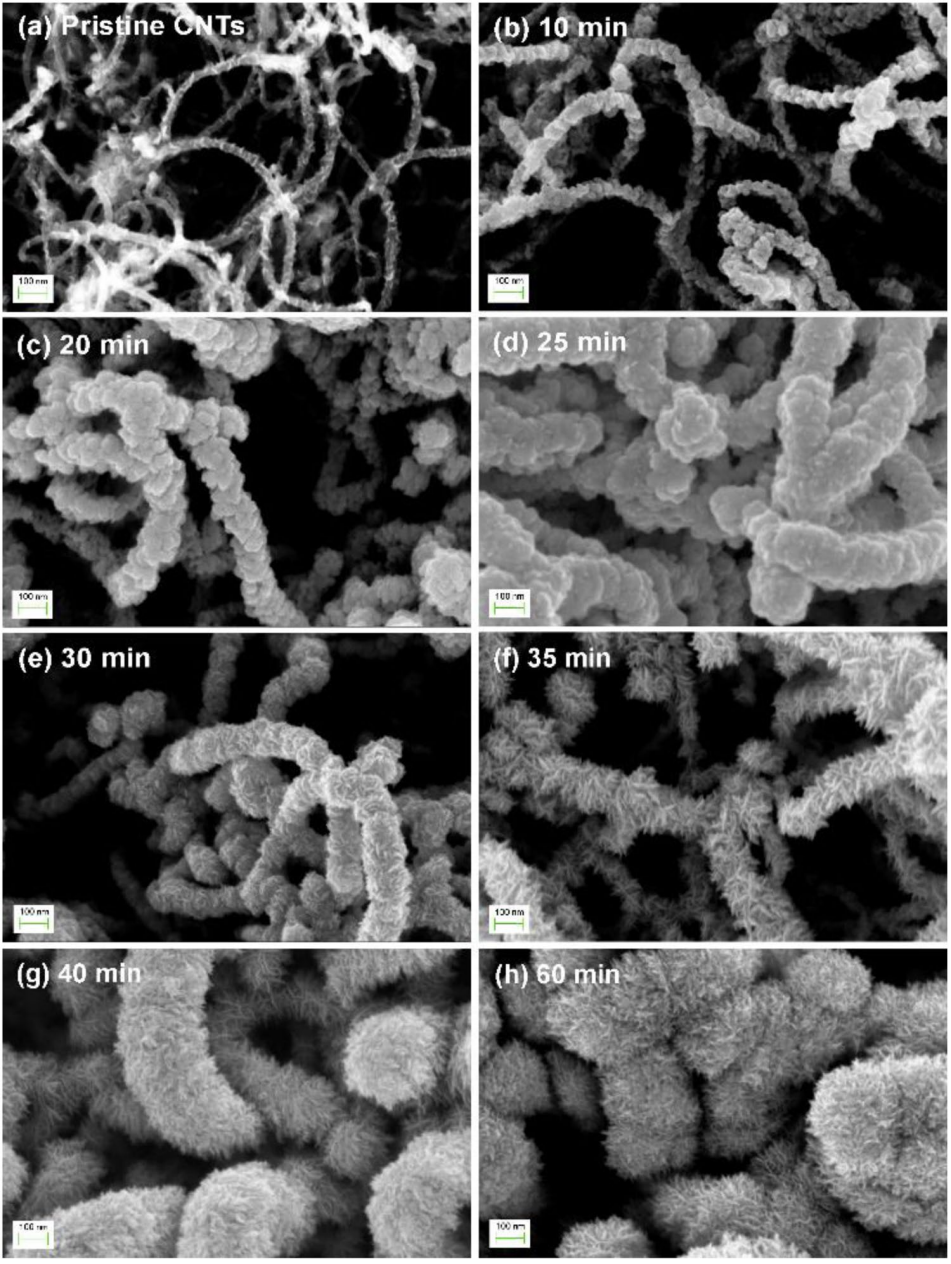
Top view SEM images of (**a**) pristine CNTs and WS_2_-CNT samples fabricated at sputter deposition times of (**b**) 10 min, (**c**) 20 min, (**d**) 25 min, (**e**) 30 min, (**f**) 35 min, (**g**) 40 min and (**h**) 60 min.

**Figure 2:**
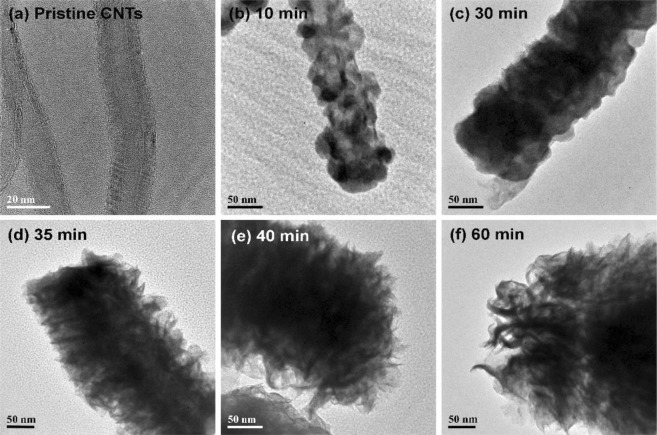
TEM images of (**a**) pristine CNTs and WS_2_-CNT samples fabricated at sputter deposition times of (**b**) 10 min, (**c**) 30 min, (**d**) 35 min, (**e**) 40 min and (**f**) 60 min.

**Figure 3:**
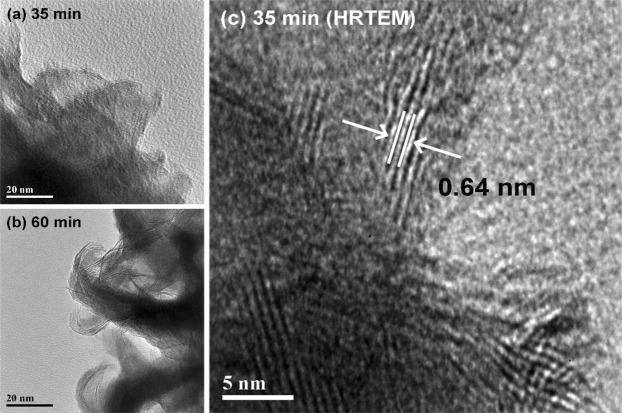
TEM images of (**a**) nano-petals in 35 min sample and (**b**) nano-bristles in 60 min sample. (**c**) HRTEM image of nano-petals in the 35 min sample showing lattice fringes of WS_2_.

The SEM images of the samples are depicted in Fig. 1, in which we observe the emergence of a film of angular particulates encapsulating the CNTs after 10 min sputter deposition of WS_2_. As the deposition time is increased, the particulate film thickens until sharp flakes begin to emerge from the surface at a sputter time of 30 min (Fig. 1(e)). These nanoflakes lengthen and increase in concentration when the sputtering time is increased to 35 min, leading to the formation of a dense forest of triangular flakes that complete envelops the CNTs. Still longer sputter times give rise to a drastic change in the sample morphology, as seen in Fig. 1(g), whereupon new nanoflakes begin to grow over pre-existing ones. These new nanoflakes appear to be much sharper and narrower, and quickly become so numerous as to give the impression of “bristles on a brush”. Any increase in the deposition times from this point forth only serves to thicken the encapsulating layer, but induces little change in the morphology of the samples. This transformation of the WS_2_-CNT hybrid nanostructures is corroborated by the TEM images of the samples, which is shown in Fig. 2. Uncoated multi-walled CNTs have diameters of approximately 20 nm (Fig. 2(a)). After sputtering with WS_2_ for 10 min, the nanotubes becomes coated with angular particulates that thicken to become a film at a sputter time of 30 min. Thin sheets of material can also be seen extending outward from the sidewalls of the encapsulated CNTs, similar to the results obtained from SEM. These thin sheets increase in density and length as sputter time is increased to form triangular nanoflakes that eventually begin stacking on top of each other. The topmost flakes appear to preferentially roll themselves into a narrow and highly curved geometry, as illustrated in Fig. 2(f). Higher magnification images in Fig. 3(a,b) gives a clearer view of the differences in morphology between the nanoflakes formed at a sputter time of 35 min versus 60 min respectively. To differentiate the two types of nanoflakes, we dub the former “petals” and the latter “bristles” in keeping with their unique shapes. Both the nano-petals and nano-bristles were found to be crystalline, with high resolution TEM images of the 35 min sample (Fig. 3(c)) exhibiting the presence of lattice fringes with interplanar spacings of 0.64 nm, which is characteristic of 2H-WS_2_ [JCPDS #08-0237].

**Figure 4:**
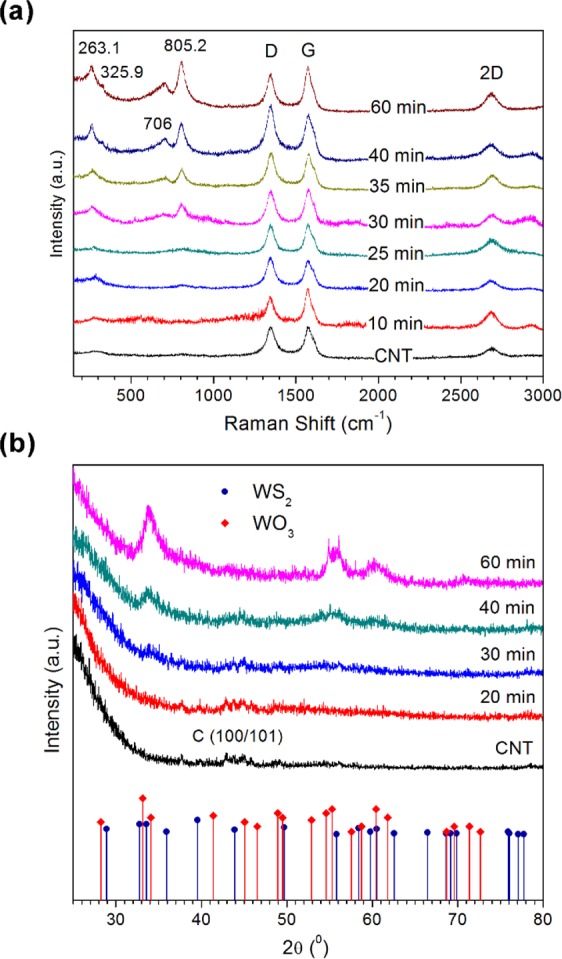
(**a**) Raman spectra of pristine CNTs and WS_2_-CNT samples. (**b**) X-ray diffractograms of WS_2_-CNT samples with bulk diffraction peaks of 2H-WS and WO_3_.

The optical properties of our as-grown samples were analysed by Raman spectroscopy and the results are depicted in Fig. 4(a). Three distinct peaks at 1350 cm^−1^, 1575 cm^−1^, and 2969 cm^−1^ were observed for all samples, corresponding to the disorder (D), graphitic (G) and second-order disorder (2D) peak of carbon. The G peak is due to the E_2g_ mode at the Γ-point^25^. It arises from the stretching of C-C bonds in graphitic material, and is common to all sp^2^ hybridized carbon systems. The D peak on the other hand corresponds to the breathing mode of six-fold aromatic rings of sp^2^ carbon and is activated by disorder in the graphitic structure^26^. The 2D band is excited by a double-resonant Raman process that, unlike the D band, is present even in the absence of defects. The carbon region of the Raman spectra remains unchanged even after prolonged deposition of WS_2_, indicating that the coating does not interact strongly with the nanotubes. At a sputter time of 20 min, we observe a small broad peak at approximately 800 cm^−1^, which gradually intensifies until two distinct peaks at 704.1 cm^−1^ and 804.9 cm^−1^ emerge at 30 min sputter time. Both peaks are matched to the W-O stretching modes in tungsten (VI) oxide^27^. A shoulder at approximately 300 cm^−1^ can also be observed in the Raman spectra at 20 min. With increasing deposition times, this feature transforms into a definite peak at 263.1 cm^−1^ and another weak shoulder appears at 325.9 cm^−1^. Both bands correspond to the O-W-O bending modes of WO_3_^27^. This trend suggests that the particulate film formed during initial deposition is composed of amorphous WO_3_ that then re-crystallizes with longer sputter times. There appears to be no contribution whatsoever from WS_2_ regardless of sputtering duration, which is curious as the TEM images depict the distinctive crystalline 2D flakes of WS_2_. We attribute this to the fact that first, WO_3_ signals are overwhelming due to the much higher concentration of the WO_3_ interface/support layer as compared with the 2D WS_2_ petals that extends from the surface. It is further noted the WO_3_ peaks at 330 cm^−1^ slightly overlaps and easily mask the much weaker WS_2_ Raman peaks at 350 cm^−1^ leading to this shadowing effect which resulted in the non-detection of the characteristic Raman modes of WS_2_.

Further analysis of the composition and crystallinity of the as-grown samples were conducted by thin film XRD (Fig. 4(b)). Two slightly distinguishable peaks at 2θ = 42.7 ° and 43.9, indexed to the (100) and (101) reflections of graphitic carbon respectively [JCPDS ##65-6212], is observed for pristine CNT and all WS_2_-CNT composites except for the sample sputter deposited at 60 min (Fig. 4(a)). At such prolonged sputtering times, the deposited coating is thick enough that incident X-rays are unable to penetrate into the CNT layer. No other peaks are observed in the XRD profile for pristine CNTs and the 20 min sample, not even from a WO_3_ phase, which tallies with the Raman results indicating that the WO_3_ particulate film is still amorphous or poorly crystalline at this sputtering time. After 30 min however, a small hump centred at 2θ = 33.9° begins to emerge, and by 60 min, it is joined by three additional peaks with broad FWHMs due to the overlapping of signals from the WS_2_ and WO_3_ phase. Despite the strong overlap, it can clearly be seen that the reflections at 2θ = 54.6°, 55.3° and 71.4° arise solely from the WO_3_ phase [JCPDS #05-0388]. The first two peaks begin to emerge at sputter times of 30 min, which matches with the Raman results indicating that crystallization of the amorphous WO_3_ phase occurs from this sputter time onwards. On the other hand, the reflections at 2θ = 33.9°, 55.8° and 60.5° can be indexed to hexagonal 2H-WS_2_ [JCPDS #08-0237], confirming its presence in the samples sputter-deposited for 30 min or longer. This corroborates the results from the SEM and TEM images, from which we observed the emergence of sharp flakes at a sputter time of 30 min.

**Figure 5:**
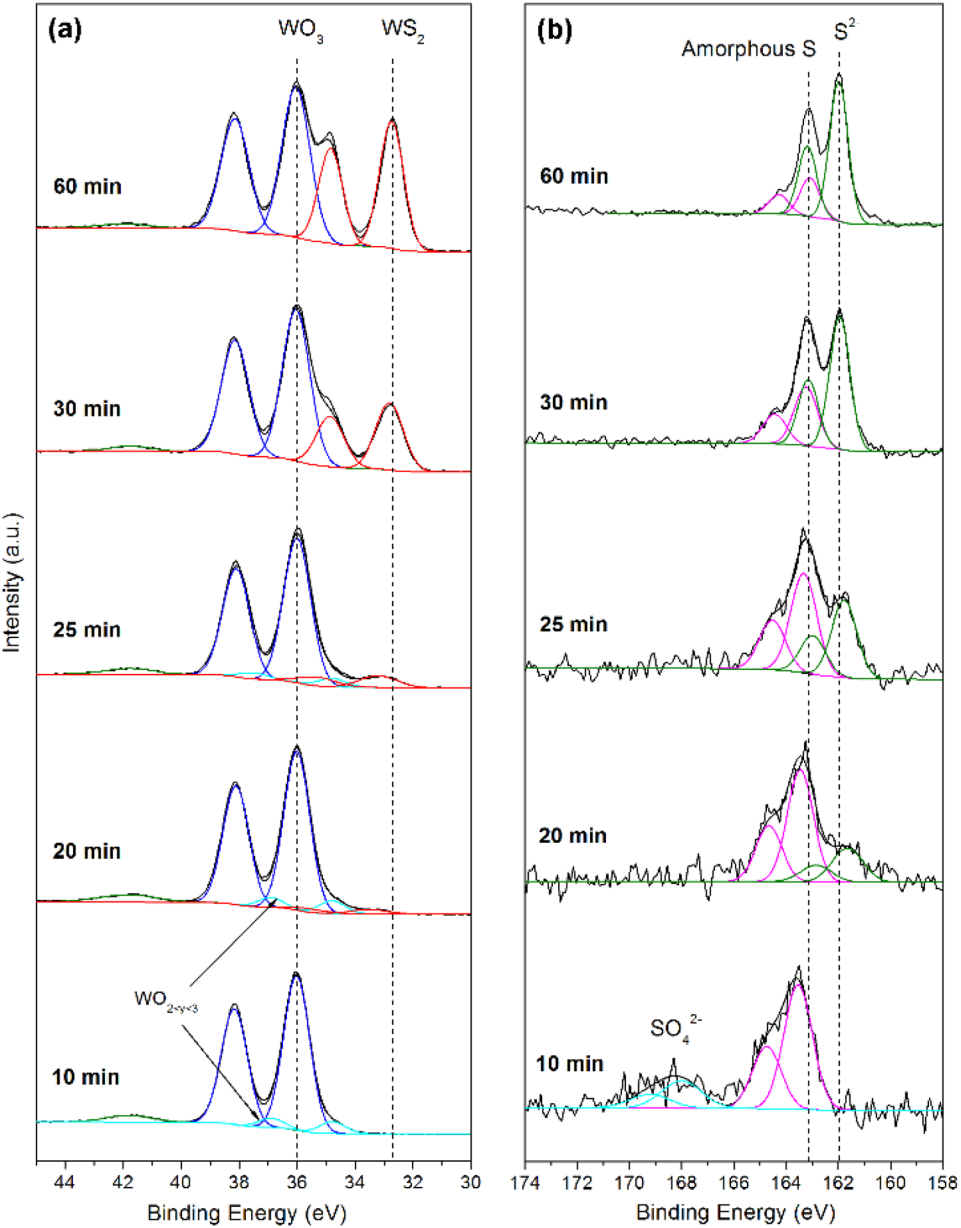
High resolution XPS scans of (**a**) W 5d and (**b**) S 2p core levels for selected WS_2_-CNT samples.

High resolution XPS scans of W 4f and S 2p core level spectra for selected samples are presented in Fig. 5. For each sample, there is a W 4f doublet at binding energies of 36.0 and 38.1 eV (blue curve) owing to the W^6+^ oxidation state of WO_3_, thus confirming the presence of the WO_3_ phase. For the samples sputter deposited at 10, 20 and 25 min, there is an additional doublet at binding energies of 34.8 and 36.9 eV (cyan curve), which can be assigned to a W^5+^ state in WO_y_ where 2 < y < 3^28^. Beginning from the 20 min sample, an third W 4 f doublet emerges (red curve). This doublet first occurs at binding energies of 33.6 and 37.7 eV, then gradually shifts to lower energies with increasing sputtering duration until it stabilizes at around 32.7 and 34.8 eV when a sputter time of 30 min is reached. These values of binding energies corresponds well a W^4+^ species in an intermediate O-W-S state that undergoes chemical change to form highly crystalline 2H-WS_2_ at 30 min sputter time. Although the relative proportions of this phase increase with sputter time, WS_2_ remains the minor compound compared to WO_3_ even at the longest sputter time of 60 min. This dominance of the oxide phase could explain the difficulty in detecting the characteristic Raman peaks of WS_2_ as previously discussed. Figure 5(b) depicts the S 2p spectra of the WS_2_-CNT samples. At 10 min sputter time, there are two S 2p doublets, one located at 163.2 and 164.4 eV due to the presence of amorphous sulfur and the other pair at 168.25 and 169.45 eV due to the oxidized sulfur species SO_4_^2−^. Although the SO_4_^2−^ vanishes at longer sputtering times, the peaks from amorphous sulfur remains present throughout all samples. From 20 min onwards, a new doublet at binding energies of approximately 161.6 and 162.8 eV begins to appear and gradually shifts to slightly higher binding energies with longer sputter times. They eventually stabilize after 30 min of sputtering at values of 162.0 and 163.2 eV, which agrees well with the S^2−^ species in WS_2_^23^. This gradual shift can be attributed to increasing electron withdrawing character of the W species bonded to S, thus complementing the chemical change we observed in the W 4 f spectra where the O-W-S bond converted to the S-W-S bonds at 30 min sputter time. These results collectively indicate that the WS_2_ phase produced at low sputter deposition times (<30 min) are highly defective and sulfur-deficient. One possible reason for this is the preference of sulfur atoms to bind to oxygen or to itself, as evidenced by the pronounced peaks from oxidized SO_4_^2−^ species and amorphous sulfur. As sputter time increases beyond 30 min however, the SO_4_^2−^ peaks disappears in conjunction with a reduction of the amorphous sulfur signal intensity relative to the S^2−^ species, all of which coincide with a downwards shift of the binding energies of the W^4+^ doublet. This behaviour suggests that longer deposition times favours the gradual displacement of oxygen atoms by sulfur in the W compound to form WS_2_.

**Figure 6:**
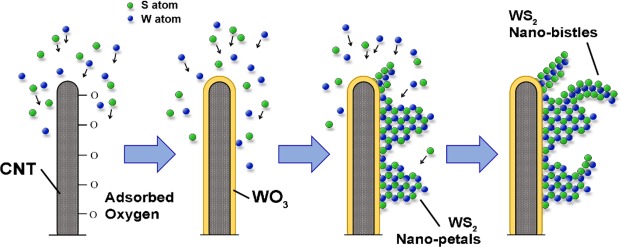
Proposed schematic growth process of WS_2_ nano-petals and nano-bristles supported on CNT.

Figure 6 shows the proposed schematic growth process of the core-shell WS_2_-CNT nanostructures. In the synthesis of WS_2_ on CNTs, no external catalysts or metal seeds were involved, which makes the process simple and clean. The lack of catalysts or seeds also suggests that a modified vapour-solid (VS) growth mechanism is pre-dominant instead of the typical vapour-liquid-solid (VLS) growth processes^29,30^. In the sputtering process, W and S atoms are removed from a solid target by bombardment with high energetic ions that imparts high mobility to the source atoms, allowing them to quickly reach the CNTs and move along the surface of the nanotubes despite the lack of heat supplied. The source atoms are attracted to the defective areas in CNTs, which due to their higher reactivity compared to the surface plan regions, act as favourable sites for nucleation and subsequent crystal growth. In PECVD grown CNTs, these defects can take the form of “cross-struts”, pentagon-heptagon pairs, vacancies and ion impurities^31^. Other important defects include grain boundaries in the graphene layers of the nanotube wall that can act as nucleation sites due to the presence of dangling bonds that attract adsorbates^32^.

In the initial growth process, the W and S atoms that reach the surface of the substrate do not interact with each other. Instead, the presence of adsorbed oxygen on the CNT walls leads to significant oxidation of the W and S species to form an amorphous WO_3_ base layer with adsorbed gaseous SO_4_^2−^ species. Excess S atoms then bind to each other to form amorphous sulfur. Once most of the oxygen is consumed and the concentration of S atoms at the surface reaches a critical value (sputter time >10 min), sulfurization of the as-formed WO_3_ phase begins to take place. The process is most likely analogous to the CVD growth of 2D WS_2_ layers involving the reaction of sulfur vapour with a pre-deposited WO_3_ thin film at high temperatures^24,33^. In our samples, we are able to determine that initial sulfurization proceeds via the displacement of oxygen atoms in WO_3_ with S atoms to form, first O-W-S bonds, and then finally S-W-S. These clusters of pure WS_2_ crystals nucleate on top of the oxide layer, with kinetics initially favouring the lateral growth of WS_2_ islands due to minimization of energy. This can be deduced from the layered structure of WS_2_, wherein the surface energy of the planes perpendicular to the *c*-axis is much smaller than others due to the weak van der Waals forces holding the layers along this axis together. However, the rate at which the WS_2_ nuclei grow is much slower than the rate at which W and S atoms reach the surface of the oxide layer. Consequently, supersaturation of the vapour occurs and induces fresh WS_2_ nuclei to quickly stack on top of previously formed islands along the direction perpendicular to the CNT surface, resulting in vertically standing, triangular-shaped nanoflakes reminiscent of flower petals. These nano-petals proliferate with increasing sputter time until a critical density is reached at 40 min. At this point, the nano-petal forest encapsulating the nanotube is too dense to allow additional flakes to develop as quickly as during the initial stage of sputtering. The continued transport of source atoms at the same rate thus leads to the vapour conditions around the nano-petals becoming even more supersaturated, triggering secondary branching in a process resembling dendritic outgrowth. Incoming source atoms thus begin to form nuclei and islands on the surface of the nano-petals. TMD layers are known to be unstable towards bending and have a high propensity to roll into curved structures^34^. Combined with the strict space constraints between each nano-petal, the secondary nanoflakes that form eventually begin to roll and twist into the curved structures with needle-sharp tips seen in the TEM images. Hence the term nano-bristles.

**Figure 7:**
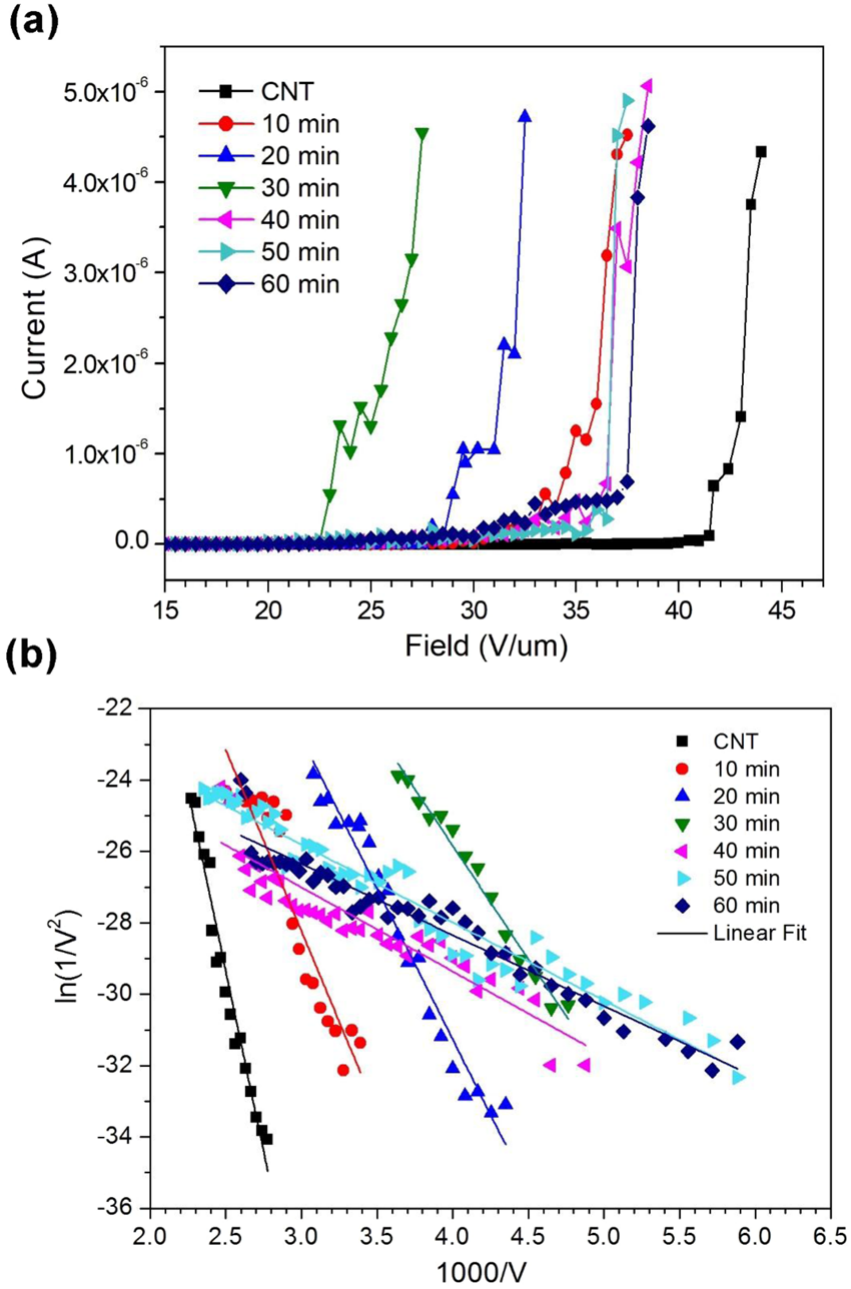
(**a**) Field emission plots of current versus voltage and (**b**) Fowler-Nordheim plots for WS_2_-CNT samples.

In order to investigate the field emission properties of the films, I-V curves were obtained with a probe tip setup at an anode-to-cathode distance of 10 μm. Figure 7(a) shows the I-V plots of selected WS_2_-CNT samples. The turn-on voltage, defined as the voltage required to produce a current of 1 μA, is determined to be 42.5 V/μm for pristine CNTs used in this work. For the 10, 20, 30, 40, 50 and 60 min samples, the measured turn-on voltages are 34.8, 29.5, 23.2, 35.5, 35.6, and 37.6 V/μm respectively, demonstrating an improvement in field emission performance that peaks at the 30 min sample. We have to be careful in the comparison here as the same pristine CNTs were found to have a turn-on field of 5.6 V/μm in a large parallel plate capacitor setup^35^. As the tungsten probe tip emitters tend to be much more resistive and not subjected to uneven heights in the specimens^36^, our main comparison would be between the reference CNTs. In general, these results indicate that both the WO_3_ coating and WS_2_ nanoflakes are capable of enhancing the ability of CNTs to emit electrons. In addition, that the turn-on voltage increases beyond a sputter time of 30 min despite there being no change in chemical state suggests that morphological transformation is responsible for the diminishing field emission performance in these samples. Fowler-Nordheim (F-N) plots of the samples were also obtained and shown in Fig. 7(b), revealing a linear trend between $\mathrm{ln}(\frac{I}{{V}^{2}})$ and $(\frac{1}{V})$ that indicates that the electron emission occurs through a quantum tunnelling process. In addition, the observed current saturation phenomenon with a smaller line slope of each plot at the high voltage region has been known to be caused by adsorbates on the emitter surface^37^. The slope of the linear region of the F-N plot is a function of the work function of the material, *ϕ*, the field enhancement factor, *β*, and the distance between the electrodes, *d*, which can be expressed by transformation of the F-N equation as,

$$Slope=-\,\frac{6.83\times {10}^{3}{\varphi }^{\frac{3}{2}}d}{\beta }$$

Assuming *φ*_*CNT*_ = 5.0 *eV*^17^, ${\phi }_{W{O}_{3}}=4.8\,eV$^24^, and ${\phi }_{W{S}_{2}}=5.1\,eV$^33^, the value of *β* for pristine CNTs and samples 10 to 60 min is to be 39, 71, 85, 124, 334, 360 and 395 respectively.

It is widely known that field emission is a quantum mechanical tunnelling phenomenon, where electrons are emitted from a solid surface to vacuum due to the presence of an external electric field. Due to field lines converging at sharp edges and points, the presence, morphology and density of any protrusions or sharp edges on the material surface are crucial factors that affect the field emission intensity^37^. For example, in CNT arrays, electrons are transported along the nanotubes and emitted only from the tips because the flat nanotube side walls produce low local field strength. However, if additional structures are present on the outer wall of the nanotubes, additional emission sites are created that allow electrons to be emitted from the CNT side walls. This significance of the physical geometry and dimensions of emitters is reflected in F-N equation through the inclusion of the field enhancement factor, *β*. For the sputter coated CNTs in this work, all demonstrated an enhancement in *β* as compared to pristine CNTs, indicating that all three different morphologies of a WO_3_ particulate film, WS_2_ nano-petal forest, and WS_2_ nano-bristle forest provide additional sites for electron emission compared to the uncoated nanotubes. In particular, the samples with the nano-bristle morphology (40, 50 and 60 min) exhibit the highest *β* values, which is not surprising considering that they have both a higher density of sharp edges and tips as well as smaller average tip diameter compared to the nano-petals due to rolling of the flakes into the bristle geometry. What is unusual however, is that despite the having the highest *β* values, these samples also have the lowest turn-on fields of the coated CNTs. One possible reason for this is poor electrical contact between the nano-petals and nano-bristles when the latter stacks on top of the former. This would cause a substantial voltage drop to occur between the nano-petals and nano-bristles at large emission currents because of the large contact resistance, leading to current saturation. The thicker coating could also result in greater electron scattering, reducing the efficiency of electron transportation within the nanoflakes. The end result is a field performance that is comparable to the sample with WO_3_ nanoparticle coated CNTs (10 and 20 min).

**Figure 8:**
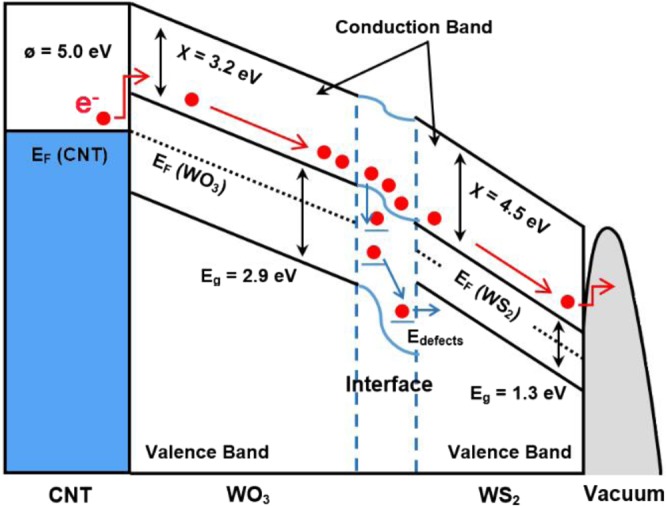
Schematic diagram of the CNT/WO_3_/WS_2_ heterojunctions under an applied electric field.

Aside from the improvement in field enhancement factor of the emitters, it is believed that the lower turn-on voltages for the composite nanostructures are also correlated the decrease in potential energy barrier because of band bending and the presence of defects at the interface between WO_3_ and WS_2_. A schematic energy band diagram of the CNT/WO_3_ and WO_3_/WS_2_ heterojunction under an applied electric field is shown in Fig. 8. Owing to the fact that the band gap of CNTs is quite narrow, about a few hundred meV at room temperature^38^, whereas WO_3_ is an n-type semiconductor with a band gap of 2.6–2.9 eV^39^, it is assumed that the heterojunction formed is similar to a metal/semiconductor junction. Consequently, the electrons are injected from the Fermi level of CNTs into the conduction band of WO_3_ by tunnelling through the Schottky barrier and then emitted from WO_3_ into vacuum. WO_3_ has a smaller electron affinity of 3.2 eV^40^ as compared to the value of 4.8 eV for CNTs^41^. This leads to much lower energy threshold for the electrons escaping from the conduction band of WO_3_ into vacuum, and thus they are more easily emitted from WO_3_ than CNTs. For samples 30 to 60 min wherein a layer of WS_2_ nanoflakes/petals grows over WO_3_, the electrons would tunnel further into the conduction band of WS_2_. The bandgap of WS_2_ has been reported to be 1.3 eV with an electron affinity of 4.5 eV^42,43^. As such, the bending band at this junction is beneficial to the movement of electrons from the WO_3_ to WS_2_ and holes from WS_2_ to WO_3_, which effectively reduces the recombination of electron-hole pairs^44^. If vacancies and defects are present at the interface, they can induce defect energy bands (E_defects_) between the energy bands of WO_3_ and WS_2_. The electrons on the WO_3_ conduction band can thus easily jump into these defect energy bands and then jump further into the WS_2_ bands. Hence, a large number of electrons gather on the surface of WS_2_ nanoflakes and are easily emitted into the vacuum through subsequent F–N tunnelling.

## Conclusion

Sputter deposition was used to fabricate core-shell WS_2_-CNT heterostructures at room temperature. An amorphous WO_3_ particulate film was initially formed over the nanotubes. However, increasing the amount of source atoms eventually results in sulfurization of the WO_3_ film to form intermediate O-W-S bonds before complete transformation to S-W-S bonds take place. The crystalline WS_2_ nanostructures on CNT take the form of vertically standing nano-petals for the 30–35 min samples, while in the 40–60 min samples they adopt a rolled and curved geometry (nano-bristles). All three morphologies of a WO_3_ particulate film, WS_2_ nano-petals and WS_2_ nano-bristles result in enhanced field emission performance over that of uncoated CNTs. The lowest turn-on voltage of 232 V was obtained for the 30 min sample. This is attributed to the combined effects of the formation of a Schottky at the CNT/WO_3_ interface, the possible presence of defects at the WO_3_/WS_2_ interface, and the geometric enhancement of sharp-tipped nano-petals that act as additional emission sites. The observed increase in turn-on voltages of the 40–60 min samples despite the much higher field enhancement factors is deduced to be due to poor electrical contact between the nano-petals and nano-bristles. The large voltage drop across this interface at high emission currents results in current saturation and thus poorer field emission performance. The successful synthesis of such hybrid structures open up a range of possibilities for new materials for nanodevices.
